# Zambia’s National Cancer Centre response to the COVID-19 pandemic—an opportunity for improved care

**DOI:** 10.3332/ecancer.2020.1051

**Published:** 2020-05-28

**Authors:** Dorothy C Lombe, Catherine K Mwaba, Susan C Msadabwe, Lewis Banda, Maurice Mwale, George Pupwe, Paul Kamfwa, Mulape Kanduza, Harry Munkupa, Biemba Maliti, Kalyoka Simbeye, Pious Hachizo, Lilie Lin, Elizabeth Chiao, Kennedy Lishimpi

**Affiliations:** 1 Department of Oncology, Cancer Diseases Hospital, Lusaka 10101, Zambia; 2 Ministry of Health Zambia, Lusaka 10101, Zambia; 3 Department of Gynaecology Oncology, Cancer Diseases Hospital, Lusaka 10101, Zambia; 4 Department of Medical Physics, Cancer Diseases Hospital, Lusaka 10101, Zambia; 5 Department of Radiation Therapy, Cancer Diseases Hospital, Lusaka 10101, Zambia; 6 Department of Nursing, Cancer Diseases Hospital, Lusaka 10101, Zambia; 7 Pharmacy Unit, Cancer Diseases Hospital, Lusaka 10101, Zambia; 8 Department of Radiation Oncology, MD Anderson Cancer Center, University of Texas, TX 77030, USA; 9 Department of Epidemiology, MD Anderson Cancer Center, University of Texas, TX 77030, USA; a https://orcid.org/0000-0002-5083-1801

**Keywords:** COVID-19, cancer centre preparedness

## Abstract

The COVID-19 pandemic has overwhelmed health systems around the globe even in countries with strong economies. This is of particular concern for nations with weaker health systems. This article reports the response of a comprehensive cancer centre in a lower-middle income country to prevent COVID-19 transmission and how the implementation of pragmatic strategies have served as a springboard to improve cancer services beyond the COVID-19 pandemic. The strategies included establishment of a local taskforce, increased education and facilitation of good hygiene practices, staff training, patient triaging, improved patient scheduling, remote review of patients and establishing a virtual platform for meetings.

## Introduction

The COVID-19 pandemic has overwhelmed health systems around the globe, prompting several large healthcare organisations and societies to issue guidelines to help prioritise the various aspects of cancer care to mitigate its negative effects [1–5]. Their main intent is to reduce transmission risks without denying access to those who need services the most, while ensuring the safety of patients, hospital staff and the public. Although helpful as a general approach to triaging care in the midst of the pandemic, most do not take into consideration some of the unique aspects of delivering cancer care in resource-constrained environments of low- and middle-income countries, and the populations they serve.

## Background

Zambia is a landlocked, lower-middle income country (gross domestic product per capita US$1539.90) located in the south-central region of Sub-Saharan Africa with a population of approximately 17 million [6, 7]. Its national cancer centre—Cancer Diseases Hospital (CDH) was established in 2006. Located in the capital city of Lusaka, it is the only centre in the country offering radiotherapy and serves the entire population of Zambia as well as some neighbouring countries (Malawi, Democratic Republic of Congo and Zimbabwe). CDH offers comprehensive cancer care and is equipped with three teletherapy machines (one Linear Accelerator and two Cobalt machines), two brachytherapy suites (one with functioning 6-channel and the other with 18-channel iridium-based high dose rate unit), two CT simulators (1 used more for diagnostic purposes), one MRI machine, Mammography, ultrasound and a digital X-ray unit. It also has a self-contained chemotherapy suite, operating theatre and a 252 in-patient bed unit. There are 7 radiation oncologists, 5 medical physicists, 25 radiation therapy technicians and 189 nurses in different capacities (12 oncology nurses, 1 palliative care nurse, 2 operating theatre nurses, 5 diagnostic nurses, 147 registered nurses, 19 enrolled nurses and 13 in administrative positions). The facility has enrolled and provided care for an increasing number of patients since its inception, totalling just above 3,000 new patients in 2019 [8]. Cervical cancer is the leading cancer with an incidence of 66.4 per 100 000, followed by prostate cancer (45.6 per 100 000) and breast cancer (19.9 per 100 000) [9].

Over 90% of the new cases that register for care at CDH present in a late stage and the overwhelming majority are suffering from severe symptomatology. A high percentage of patients travel long distances from rural areas of the country. Almost all are in the lowest income bracket. Because of these and other circumstances, deferment of cancer management services (counselling, treatment, palliation, blood transfusion, pain relief, nutritional assessment, etc.) must be avoided at all costs. In the same light, opportunities for surgical treatment of early stage cancers, particularly those originating in the breast, cervix and vulva, should not be missed as these are cases associated with high cure rates and may reduce the financial and utilization burden on radiotherapy machines as well as save on limited supplies of chemotherapy drugs [3, 10].

The existence of only one cancer centre within the country, coupled with the high burden of disease, makes the continuation of cancer care in the face of the COVD-19 pandemic an absolute necessity. Its autonomy and capacity as described above, allowed for adjustments in order to mitigate the impact of the pandemic and represent a silver lining of opportunities in a crisis to improve the quality of care. Our articulation of this juxtaposition forms the basis of this paper.

## Government response

The country commenced its response on 30th January 2020 and declared the outbreak on 18th March 2020, after recording the first two cases of COVID-19 from citizens who had returned from a holiday in France. At the time of writing this article it had 252 confirmed cases 112 recoveries and 7 deaths [11]. A cumulative number of 12,852 high risk persons were observed and 12,095 tests done with 711 tests per million population being conducted [11].

The response of the central government has been pragmatic, with emphasis on safety measures that can be initiated at the population and individual levels. The public has been kept abreast of the local status of the trend of the disease outbreak through regular daily press briefings with the Minister of Health, during which time questions from the press corps are entertained. In formulating this approach the economic, cultural and social peculiarities of the country were taken into consideration, as well as institutional experience with prior infectious disease outbreaks involving cholera, tuberculosis and Human Immunodeficiency Virus, to maximise the effectiveness of the public health response.

Zambia did not go into complete lockdown but two statutory instruments were enacted by the Zambian government on the 14th of March, 2020 [12–14]. One designated COVID-19 as a notifiable disease and the other provided for additional regulations to facilitate its management and control. The additional regulations included a restriction on all foreign travel, mandatory screening and quarantine of international travellers, heightened hygiene practices to promote infection prevention, restriction of mass gatherings, increased physical distancing and the mandatory use of face masks in all public spaces. To try and decrease transmission, services were categorised into those that are essential and non-essential. Those deemed non-essential, e.g., bars, restaurants and shopping malls were closed. Learning institutions (schools, colleges and universities) were also closed with the view to institute on-line learning. Those deemed as essential, such as health clinics and hospitals, have been allowed to maintain operations.

## Cancer centre response

### Establishment of a hospital level COVID-19 task force

Due to the above circumstances and concerns, the cancer centre leadership decided to establish a local response taskforce that could formulate, implement, enforce and communicate national measures to maintain the safety of patients and hospital staff. This task force included both clinical and administrative staff. Additionally, each unit was invited to nominate a senior staff member with managerial exposure in their day-to-day activities as well as a junior staff member who was on the treatment floor actively on a day-to-day basis to ensure solutions and strategies were practical.

### Basic epidemiologic measures

The following action as prescribed by the World Health Organisation and national leadership were implemented [2, 15]. These include the following:

1. Increasing the capacity for hand washing, sanitising and social distancing.
  - In addition to the existing water infrastructure, locally innovated mobile water reservoirs with taps were placed at strategic points all around the institution.
  - Security staff received enhanced orientation to ensure increased utilisation of the hand washing facilities.
  - The local pharmacy team reconstituted a cost effective alcohol-based hand rub as the financial barrier to commercially produced and procured one was recognised [16].
  - The environmental team placed additional sanitizer dispensers.
2. Housekeeping personnel were requested to strengthen adherence to cleaning schedules
3. Social distancing was enforced in waiting areas. For instance, a bench outside the laboratory area (a frequent stop for most patients) was clearly marked for practical demonstration as to what was an acceptable distance to lower the risk of person-to-person transmission

### Staff training

The National Response Team organised staff training, helping to ensure that all staff were adequately trained in the prevention of COVID-19 transmission. Training topics consisted of understanding the disease, how it is spread and what can be done to prevent transmission. Emphasis was placed on how to respond if faced with patients undergoing treatment who were COVID-19 positive. COVID-19 positive patients are lodged at two central quarantine centres in Lusaka, located 10 km away from the cancer centre and would attend the cancer centre as out-patients. Patients that are critically ill due to the COVID-19 will not be eligible for active cancer treatment as per standard protocol due to poor performance status score and this will be no exception during this pandemic.

### Patient triage

The access into the cancer centre has been restricted. The outpatient nurses and environmental health officers screen the attendees for raised temperature and symptoms. The outpatients with a good performance status are seen alone; those requiring assistance are allowed one caregiver to accompany them.

A holding room has been designated for any person found to be high risk for SARS-CoV-2 infection before transfer to the designated quarantine facilities by the national surveillance and response team.

### Inpatients

Following a directive from the ministry of health, all inpatients at the cancer centre were swabbed and tested for SARS-CoV-2. The nursing staff conducted education to inpatients and their caregivers on COVID-19 to strengthen adherence to good hygiene practices. Mandatory testing is on going for all new patients being admitted. Inpatients are allowed one caregiver at their bedside. Visitation for inpatients has been suspended.

### Patient appointments

All new patients continue to be seen as a priority for establishment of a treatment plan. The geographic distances travelled by many patients, socioeconomic challenges and the lack of a formal electronic based referral system means most patients continue presenting in person. This COVID-19 crisis is being seen as an opportunity to develop a robust referral system so that cancer patients do not have to wait for treatment in Lusaka or travel to and fro across the country but instead arrive for scheduled visits.

### Patient reviews

During the first 2 weeks of the pandemic, patients on surveillance following definitive cancer treatment were called by the booking desk to reschedule follow up visits. Following the establishment of a task force, medical personnel are conducting telephonic reviews.

### Scheduling of patients on radiotherapy

Patients being treated with radiotherapy are usually seen first come first serve basis. Now patients have scheduled in time slots in order to ensure that only a specific number of patients occupy the waiting area at a particular time allowing for maximal physical distancing. The socioeconomic challenges faced by patients in accessing transport to make their appointments have been recognised and the slots are further arranged into blocks to allow some flexibility.

So far no patient requiring radiotherapy or chemotherapy has been found to be COVID-19 positive, but measures have been put in place. It is suggested that such patients be allocated slots towards the end of the treatment day to allow for thorough sanitisation of the areas after treatment and to minimise the contact with other patients. A more pragmatic approach under consideration is to have a high index of suspicion and implement a more clinical symptom-based approach to diagnosis of COVID-19 cases to determine how they should be handled in the radiotherapy area until laboratory tests prove otherwise. Also recognising the debate on sensitivities of current tests being used, it would be better to err on the side of caution than put other patients and staff at risk. Patients exhibiting symptoms but not yet tested should be isolated as described above and provided a medical mask if tolerated to lower the risk of transmitting infection as they await definitive measures from the surveillance team [17].

### Scheduling of patients receiving chemotherapy

Chemotherapy scheduling as an institution had less adjustment as allocation of days was already organised around cancer site so patient congestion was more controlled. The enforcement of social distancing in the waiting rooms and chemotherapy suite was successfully implemented.

### Radiotherapy protocols

One of the most significant, but positive, adjustments in this crisis has been the need for local oncologists to reconsider the fractionation regimens for the cancer patients undergoing treatment in order to reduce length of courses of radiotherapy [5, 18, 19] (Table 1). Previously, the paucity of locally generated evidence has necessitated leaning on the recommendations from high income nations where resources and proportions of advanced cases may not be matched with ours. In some instances, this included longer conventional fractionated protocols for radical cases or multiple fractions for palliative cases. However, this crisis has impressed upon our team the need to look to alternate evidence-based regimens available in literature [5, 18, 19]. The hard balance of maintaining effectiveness but not increasing toxicity remains the goal of treatment. Stratification and recognition of disease prognosis should be as stringent as possible.

A major challenge for most cancer patients who are severely anaemic is availability of blood for transfusion or alternatives to increase the haemoglobin value. With the closure of colleges and schools that are sources of blood donors and restricted movements, this may impact our ability to optimise patients for conventional photon radiation is dependent on an oxygenated tumour environment for the indirect mechanism of action.

### Surgical oncology services

Despite the overarching aim to avoid interruption of the pace of cancer services, the surgical oncology services are negatively impacted due to reduction of external theatre space. For example, prior to the pandemic the gynaecology oncology service that had two theatres days/week, one in the adjacent Women and New Born Hospital and the other at the cancer centre theatre. This has scaled down to only operate from CDH. This is partly because of the saturation of the anaesthetic resuscitation preparedness for COVID-19. Patients may be categorised as urgent, moderately urgent and safely delayed bearing in mind that most patients present with advanced disease and further delay or deferment may push the patients into inoperable cases. Review of evidence and adaptation of the multidisciplinary team protocols to match the reduced surgical capacity was conducted. Some suggested changes to specific cancer sites to align with reduced theatre space were as follows:

1. Vulvar cancer surgery will continue as per standard of care but may suffer a 6–8 week delay
2. Endometrial cancer low risk patients with grade 1 disease can be considered for non-surgical options, including hormonal therapy and intrauterine devices. Higher-risk disease should be considered for simple hysterectomy and bilateral salpingo-oophorectomy with adjuvant therapy^3^
3. Cervical cancer operable cases (Ib1, IB2 and IIA1) will be decided on a case by case basis
4. Ovarian cancer interval debulking will be preferred even for cases qualifying for primary debulking in the immediate period to give way for other cases that have no alternate approach to delay surgical intervention.

### Palliative care services

Palliative care services are based at the cancer centre. Hospice- and home-based care are currently not well developed. The strengthening of referral and communication systems being established during this crisis may present an opportunity to expand the service and raise awareness to build capacity of the human resource structure. Currently, only patients presenting at the centre have access to the palliative care service.

### Staff

As one of the measures at the national level, all the departments were advised to categorise staff into three schedules—Schedule A staff being essential (needing to report on a daily basis), Schedule B staff (may scale down physical presence and report on rotational basis) and Schedule C (who may work from home). All staff on the clinical team were categorised as essential workers falling into Schedule A.

The mental health of frontline workers fighting an invisible enemy is essential [20]. Linkages with the mental health team were made. Both group and individual care was offered. Continued reassurance and a platform to air concerns have been provided. Supervisors have been encouraged to disseminate knowledge and provide assurance for the floor staff. Education on personal protective equipment (PPE) to be used in various scenarios is on going. The ministry of health have produced guidelines for the type of PPE to be used during the COVID-19 pandemic [17]. Staff taking care of the routine cancer patients not suspected of SARS-CoV-2 infection should wear medical masks only. When a patient has respiratory symptoms, it is recommended that the staff use a medical mask, gown, gloves and eye-protection (goggles or face shield). For aerosol-generating procedures, a respirator N95 or equivalent is advised in place of a medical mask. The prudent use and innovation of safe reuse of PPE is essential. It is also recognised that without proper training self-infection rates may rise. Therefore, emphasis has been placed on donning and doffing of appropriate PPE. Prior to the pandemic, the nursing staff and radiotherapy technicians worked in shifts. Due to the shortage of human resource across all cadres in clinical care, creation of further shifts was explored but is not possible.

### Meetings

All clinical meetings have been shifted to the virtual platform. The gynaecology-oncology multidisciplinary team (MDT) is an example where this shift has increased efficiency and organisation. The MDT consists of clinical oncologists, gynaecology oncologists, pathologists, radiologist, radiation therapist, social worker, nutritionist and palliative care nurse. The lists of patients to be discussed are prepared before hand and forwarded to the cancer centre for registration. During the meeting, additional patients are added in real time for the group to review together. The review of imaging by the radiologist has also been made easier on this platform. The appropriate members of the team to facilitate treatment review patients that are discussed in clinic.

## Conclusion

**Figure 1.**
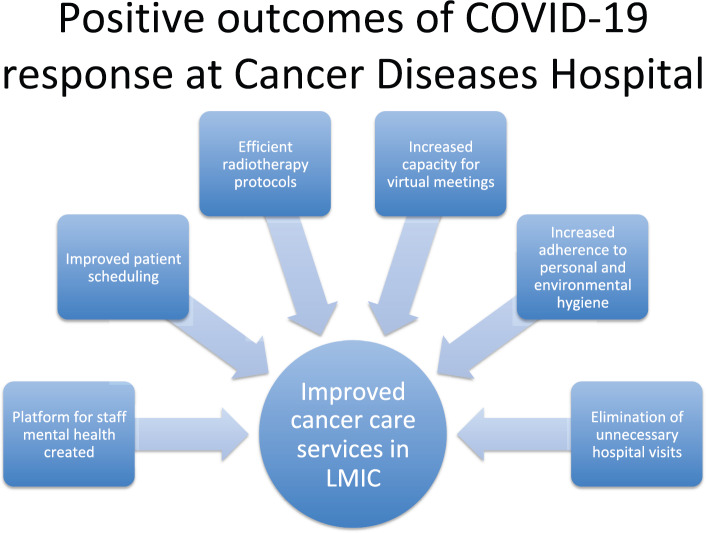
Positive outcomes of COVID-19 response at Cancer Diseases Hospital.

The COVID-19 pandemic is an ever-evolving situation. The cancer services in Zambia have taken a proactive multidisciplinary approach to continue providing care. An open communication forum (bottom up approach rather than top down) with frontline workers is essential particularly in resource-limited settings to develop effective mechanisms in order to prevent further transmission and ensure safety of patients and staff. The adjustment of workflows due to the epidemic has seen positive shifts in some aspects of service delivery for cancer patients in this low middle-income country (Figure 1). It is strongly recommended that building on these low cost changes should continue beyond the COVID-19 crisis.

## Conflicts of interest

The authors have no conflicts of interest to declare.

## Funding

This paper did not receive any funding.

## Figures and Tables

**Table 1.** Examples of proposed protocols being implemented during the COVID-19 crisis.

Site	Current Protocol	Proposed Protocol
**Radical**		
Breast Chest wall	50 Gy/ 25 #s	28.5 Gy/5 #s for 5 weeks
Breast supraclav + chest wall	50 Gy/ 25 #s	40 Gy/ 10 #s
Cervix EBRT St III bulky	50 Gy/ 25 #s	41.25 Gy/15 #s
Cervix Brachy	7 Gy x 4 #s	8 Gy x 3 9 Gy x 2 one week apart; 9.4 Gy x 2 one week apart
Prostate High risk	74 Gy/37 #s	60 Gy/ 20 #s
**Palliative**		
Breast palliative	20 Gy/5 #s	8 Gy/1#
Cervix EBRT St IVA (VVF, RVF)	41.25/15#s	10 Gy x 2 #s four weeks apart
Head and Neck palliative	30 Gy/ 10 #s	20 Gy/ 5 #s
Spinal Cord Compression	20 Gy/ 5 #s 30 Gy/ 5 #s	8 Gy/ 1#
Gy—Gray; #—fraction; Supraclav—supraclavicular; EBRT—external beam radiotherapy; ST—stage; VVF—Vesicovaginal fistula; RVF—Rectovaginal fistula; Brachy—brachytherapy.		
